# Prevalence of behavioural risk factors and their combinations among older adults: cross-sectional results of the nationwide study Gesundheit 65+

**DOI:** 10.1186/s12877-026-08007-3

**Published:** 2026-08-12

**Authors:** Beate Gaertner, Grit Eckert, Judith Fuchs, Jennis Freyer-Adam, Anne Starker

**Affiliations:** 1 Department of Epidemiology and Health Monitoring, Robert Koch Institute, Berlin, Germany; 2 Department of Prevention Research and Social Medicine, Institute for Community Medicine, University Medicine Greifswald, Greifswald, Germany

**Keywords:** Health risk behaviours, Obesity, Aged, Prevalence, Public health

## Abstract

**Background:**

Behavioural risk factors (BRFs) negatively impact health and well-being across life course, including older age. Data on their prevalence among older adults in Germany are scarce. This study examines prevalence of single BRFs — alcohol use, smoking, insufficient vegetable and fruit consumption, physical inactivity, and obesity — and identifies BRF combinations with a focus on sociodemographic disparities in adults aged 65 years and older.

**Methods:**

The nationwide *Gesundheit 65 +* study (06/2021–04/2022) used a two-stage, stratified random sample from residents’ registration offices, and a design tailored to older adults. Data from 3,139 participants with complete information on all BRFs were analysed. Weighted prevalence estimates and 95% confidence intervals (CI) for BRFs, their total number, and combinations were stratified by age, sex, and education. Non-overlapping CIs indicated statistical significance.

**Results:**

Prevalence estimates were 78.4% for physical inactivity, 71.8% for insufficient vegetable and fruit consumption, 56.3% for alcohol use, 21.0% for obesity, and 9.9% for smoking. Men reported more often than women alcohol use (69.8% vs. 45.2%) and inadequate diet (77.9% vs. 66.7%). Except for inadequate diet, prevalence varied by age and education. Participants averaged 2.4 BRFs; 83.1% reported multiple BRFs. The most frequent combinations were alcohol use + inadequate diet + physical inactivity (21.8%), inadequate diet + physical inactivity (16.0%), and physical inactivity alone (8.3%).

**Conclusions:**

Multiple BRFs are highly prevalent among older adults in Germany, highlighting the need for comprehensive strategies combining policy measures (e.g. bans on alcohol advertising), community-level interventions (e.g. improved walkability), and targeted individual interventions to promote balanced diet, physical activity, and reduced alcohol use.

**Supplementary Information:**

The online version contains supplementary material available at 10.1186/s12877-026-08007-3.

## Introduction

Among adults aged 65 years and older, chronic conditions and multimorbidity are common worldwide [1]. Most of these widespread non-communicable diseases, and related deaths are attributable to modifiable behavioural risk factors (BRFs) (e.g. [2–4]). Among those, four common behaviours, i.e. alcohol use, tobacco smoking, unhealthy diet and insufficient physical activity as well as obesity, a metabolic consequence from inadequately balanced diet and physical inactivity, are widely considered as core BRFs. Moreover, each additional BRF increases mortality risk, often more than additively [5–7]. In older age, BRFs have been found to be associated with more years lived with disability and chronic conditions as well as lower satisfaction with life and lower sleep quality [8–10]. The effect of single BRFs on the risk of chronic conditions does not significantly interact with age, indicating that the combined impact of these behaviours is substantial across all adult age groups [10].

Although the prevention of BRFs is relevant throughout the entire life-course, only few population-based studies have examined prevalence estimates and their co-occurrence in older adults. Existing studies show that, similar to the total population, about 90% of older adults report at least one of four BRFs [11, 12], and BRFs often cluster within individuals. This means that more than 50% of older adults report two to four of four BRFs [11, 13]. Unhealthy diet, insufficient physical activity and related overweight are the most common single BRFs [11, 14], and the combination of overweight and insufficient physical activity is the most common BRF combination (e.g. [11, 15]).

Population-based studies consistently show that the number and combinations of BRFs vary by demographic and socioeconomic characteristics [16]. Several studies have identified age-specific combinations in the combination of BRFs. Older age is related to higher prevalence of unhealthy diet, physical inactivity, overweight and their combination [11, 15, 17]. The prevalence of the most common BRF combination, physical inactivity and overweight, increases by 4% with each year of life [11]. Multiple BRFs and tobacco smoking occur less frequently in older age [11, 18–20]. Some studies reported lower prevalence estimates of high alcohol use in older age, while others found high prevalence estimates of daily alcohol use [18, 19]. There are well-documented sex differences in both, the prevalence and combination of BRFs. Overall, men tend to engage in more BRFs than women [20, 21]. For older adults overweight or obesity, smoking, and high alcohol use is more prevalent in men than in women, whereas physical inactivity is less prevalent in men [13].

The prevalence and the combination of BRFs are also associated with socio-economic conditions. A study found that the level of education is inversely associated with most of the 15 possible unhealthy combinations of the four BRFs examined [11]. Tobacco smoking appears to be related to low socio-economic status, and high alcohol use to high socio-economic status [11, 18, 22, 23].

The identification and prevalence of BRFs in the general population depend on current recommendations and their operational definitions. Regarding alcohol consumption, the widely used dichotomous concept of at-risk vs. not at-risk alcohol use appears to be no longer tenable. Comparable to tobacco smoking, scientific evidence demonstrates a dose-response-relationship for alcohol consumption, indicating that the amount of exposure is linked to the magnitude of health outcomes and that there is no risk-free level (e.g. [24]). In Germany, a country with high per capita alcohol consumption, national guidelines have recently abolished low risk drinking thresholds. The new recommendation advises people to reduce alcohol intake regardless of their previous consumption level, with abstaining from alcohol entirely considered optimal [25]. In view of the decline in alcohol tolerance and the high prevalence of chronic conditions in older people, as well as the potential for dangerous interactions when combined with medication [26], it appears particularly appropriate to apply more rigorous criteria to the identification of BRF alcohol use in this age group.

The aims of this study are threefold: (1) to report prevalence of single BRFs (i.e. alcohol use, smoking, insufficient vegetable and fruit consumption, physical inactivity, and obesity) among older adults in Germany, (2) to identify BRF combinations, and (3) to report prevalence stratified by sex, age groups and level of education.

## Methods

### Study design

The design of this nationwide population-based epidemiological Study on Health of Older People (Gesundheit 65+) has been described in detail elsewhere [27]. The target population of the study were German speaking persons 65 years and older permanently living in Germany, with the aim of reporting on the health status of old and very old adults in Germany. A two-staged random sampling procedure was applied: (1) nationwide municipalities were selected by random, and (2) disproportional sex- and age-stratified random samples of persons aged 65 to 79 years and 80 years and older were selected from local population registers. Apart from those who had died/ moved before the field period started or were untraceable, only those with insufficient knowledge of the German language were excluded from the study. Participation was possible regardless of the health status of the invited person. If necessary, assistances from others, proxy participation, and informed consent from legal representatives were allowed. Data was collected from June 2021 to April 2022 by self-administrated paper-based/ online questionnaires or telephone/ face-to-face interviews.

A total of 12,248 individuals were selected by random from the local registers. Only 307 individuals met the exclusion criteria described above, leaving 11,941 individuals eligible for participation with 7,706 aged 80 years and older. Of all eligible individuals, 3,694 participated in the study, including 327 participations by proxy-report. This corresponded to an overall response rate of 30.9%, calculated according to the ‘Standards of the American Association for Public Opinion Research’ [28] and including partial participation. The response rate was higher among adults aged 65 to 79 years than among those aged 80 years and older (36.0% vs. 28.1%). Of all participants, 3,139 provided complete information on five BRFs and were included in the present analyses.

### Measures

Five single BRFs were assessed: alcohol use, smoking, inadequate diet, physical inactivity, and obesity. Questions were selected from other studies conducted by the Robert Koch Institute to facilitate comparative analyses.

Alcohol use was recorded in terms of frequency and quantity. First, participants were asked, ‘In the past 12 months, how often have you had an alcoholic drink?’ with three response categories: more than once a month; once a month or less often; never [29]. If participants reported any alcohol use, the total number of standard drinks (~ 11.5 g of pure alcohol) consumed in the past week was assessed [30]. As only complete abstinence from alcohol is considered risk free [25], any alcohol use in the past week was defined as BRF *alcohol use*.

Smoking status was assessed using the question ‘Do you smoke?’ with four response categories: yes, daily; yes, occasionally; no, not anymore; I have never smoked [31]. Any *smoking* included daily and occasional smoking and was defined as BRF *smoking*.

As one aspect of diet, the frequency of vegetable and fruit consumption was assessed by two questions: (1) ‘How often do you eat vegetables or salad? Please include dried, frozen and canned vegetables, but not potatoes or vegetable juice’, and (2) ‘How often do you eat fruit? Please include dried, frozen and canned fruit, but not fruit juice’. Five response options ranged from ‘once or more a day’ to ‘never’ [29]. Non-daily consumption of vegetables *and* fruits was considered as BRF *inadequate diet*.

Physical activity was assessed by a frequency-quantity measure and refers to the last three months. First, participants were asked, ‘On how many days a week are you physically active in a way that you start to sweat or get out of breath?’ Then, they were asked, ‘And how long are you physically active on average on the days when you start to sweat or get out of breath due to your physical activity?’ with four response categories: less than 10 min; 10 to less than 30 min; 30 to less than 60 min; more than 60 min [32]. To calculate the total minutes of physical activity per week, the midpoint of these answer categories was used for the quantity component, and 60 min was used for the highest category. According to the World Health Organization’s (WHO) guideline [33], less than 150 min per week were considered as BRF *physical inactivity*.

Overweight was assessed using self-reported body height (in cm) and body weight (in kg) [29]. Body mass index (BMI) was calculated as weight divided by height squared (kg/m²). According to the WHO [34], values of ≥ 30 kg/m² were deemed as BRF *obesity*.

To analyse multiple BRFs and BRF combinations, we created three variables: (1) the total number of BRF number (sum score of all present BRFs ranging from 0 to 5) (2), the presence of multiple BRFs (≥ 2 BRFs), and (3) BRF combinations comprising all 32 possible combinations of the five BRFs (31 unhealthy combinations and one healthy combination).

We considered sex (women, men), age groups, and education level as stratification variables. Two age groups were defined: 65 to 79 years old and 80 years and older. The Comparative Analyses of Social Mobility in Industrial Nations classification (CASMIN) [35] was applied to categorise participants by their highest level of school and vocational/ professional education attained into three levels: low (primary and low secondary education), medium (intermediate and high secondary education), and high (tertiary education).

### Statistical analyses

Descriptive statistics (prevalence and their 95% confidence intervals, CI) were calculated for single and multiple BRFs, as well as BRF combinations, in the total sample and by sex, age group, and education level. A difference between groups was assumed when the CIs did not overlap.

A weighting factor was calculated in order to correct the prevalence estimates for deviations between all study participants (*n* = 3,694) from the target population of people aged 65 years and older in Germany as of 31st December 2020 arising from sampling and non-response bias. The weighting factor accounted for differences in sex, age, region of residence, municipality size and level of education [27].

Two sensitivity analyses were conducted. First, we assessed the potential impact of missing information on the prevalence of single BRFs. We quantified the amount of missing data and compared prevalence estimates for the total sample and selected sociodemographic groups using alternative approaches to handling missing data. In addition to the complete-case analysis, we repeated the analyses allowing one missing BRF and using available-case analyses based on all valid observations for each BRF. The resulting prevalence estimates were compared across approaches. Second, to assess the potential impact of proxy participation, we repeated the complete-case analysis after excluding participants with proxy-report. Prevalence estimates for the total sample and selected sociodemographic groups were compared with those from the main analysis. All analyses were performed using Stata/SE Version 17.0 (Stata Corp., College Station, TX, USA, 2017). To appropriately consider weighting in calculating confidence intervals, all analyses were calculated using the survey procedures of Stata 17.0. Cases with missing values were deleted listwise.

## Results

### Sample description

Of the total sample, 47.3% were women, 57.4% were aged 80 years or older, and 47.4% had a low level of education (unweighted analyses, see Table 1).

**Table 1:** Sample description (*n* = 3,139)

	Missing values	Total		Women		Men	
	*n*	*n* (% unweighted)	% weighted	*n* (% unweighted)	% weighted	*n* (% unweighted)	% weighted
Sex	0						
Women		1,484 (47.3)	54.8	-	-	-	-
Men		1,655 (52.7)	45.2	-	-	-	-
Age in years	0						
Mean		78.6	75.5	78.9	76.2	78.3	74.6
SD		7.5	-	7.8	-	7.2	-
Range		65–100	-	65–100	-	65–100	-
Age groups in years	0						
65–79		1,337 (42.6)	68.9	612 (41.2)	65.2	725 (43.8)	73.4
80+		1,802 (57.4)	31.1	872 (58.8)	34.8	930 (56.2)	26.6
Education level	32						
Low		1,474 (47.4)	52.5	771 (52.3)	57.1	703 (43.0)	46.9
Medium		862 (27.7)	32.8	463 (31.4)	33.4	399 (24.4)	32.1
High		771 (24.8)	14.7	239 (16.2)	9.5	532 (32.6)	21.1

*SD* Standard deviation; due to rounding, the percentages do not add up to 100.0%

### Single behavioural risk factors

In descending order of frequency, BRFs in the total sample were physical inactivity (78.4%) and inadequate diet (71.8%), followed by alcohol use (56.3%), obesity (21.0%) and smoking (9.9%, Table 2).

**Table 2:** Prevalence of single behavioural risk factors overall and by sex (%, 95% confidence intervals, weighted analyses)

	Alcohol use	Smoking	Inadequate diet	Physical inactivity	Obesity
Total	56.3 (53.8–58.8)	9.9 (8.4–11.6)	71.8 (69.3–74.2)	78.4 (75.7–80.9)	21.0 (19.0-23.2)
Age groups in years					
65–79	60.6 (57.5–63.5)	12.9 (10.7–15.4)	73.4 (70.2–76.4)	73.8 (70.2–77.2)	22.7 (20.0-25.5)
80+	47.0 (43.3–50.7)	3.2 (2.5–4.1)	68.1 (65.0-71.1)	88.5 (86.5–90.3)	17.4 (15.3–19.8)
Education level					
Low	48.2 (44.7–51.8)	8.6 (6.9–10.6)	72.1 (68.7–75.3)	80.8 (76.6–84.3)	23.1 (20.3–26.2)
Medium	60.2 (56.0-64.3)	13.7 (10.6–17.5)	71.8 (67.2–76.0)	77.3 (73.0-81.2)	19.6 (16.0-23.9)
High	77.0 (73.2–80.3)	6.6 (4.4–9.8)	70.3 (65.1–75.0)	71.3 (66.7–75.5)	16.3 (13.2–20.1)
Women	45.2 (41.4–49.1)	8.7 (6.9–11.0)	66.7 (63.2–70.1)	80.8 (77.3–83.8)	21.2 (18.4–24.2)
Age groups in years					
65–79	50.8 (45.7–55.8)	12.3 (9.5–15.8)	67.5 (62.8–72.0)	75.9 (71.2–80.1)	22.2 (18.3–26.6)
80+	34.8 (30.2–39.7)	2.0 (1.2–3.2)	65.2 (60.8–69.3)	89.9 (86.8–92.3)	19.3 (16.3–22.7)
Education level					
Low	36.3 (31.8–41.0)	8.5 (6.1–11.6)	70.1 (65.6–74.3)	83.8 (79.3–87.4)	23.7 (19.9–27.9)
Medium	54.0 (47.6–60.3)	10.5 (7.2–15.1)	63.1 (56.7–69.0)	77.5 (71.5–82.6)	18.0 (13.7–23.3)
High	69.5 (61.4–76.5)	4.9 (2.6–8.9)	57.5 (48.1–66.3)	72.7 (65.3–79.0)	15.6 (10.3–22.9)
Men	69.8 (66.5–72.9)	11.3 (9.0–14.0)	77.9 (75.0-80.5)	75.6 (71.4–79.3)	20.9 (18.2–23.9)
Age groups in years					
65–79	71.1 (67.0-74.9)	13.5 (10.5–17.2)	79.7 (75.9–83.1)	71.6 (66.2–76.6)	23.2 (19.8–26.9)
80+	66.2 (62.3–69.9)	5.0 (3.7–6.8)	72.8 (69.0-76.3)	86.4 (83.5–88.9)	14.5 (11.8–17.8)
Education level					
Low	66.1 (61.1–70.7)	8.9 (6.5–12.0)	75.1 (70.2–79.4)	76.3 (69.4–82.0)	22.3 (18.5–26.6)
Medium	68.2 (61.1–74.6)	17.7 (12.7–24.3)	83.0 (77.3–87.5)	77.1 (70.6–82.5)	21.7 (16.6–27.8)
High	81.1 (76.5–85.0)	7.5 (4.7–11.9)	77.4 (72.9–81.3)	70.5 (65.1–75.5)	16.8 (13.0-21.4)

No sex differences were observed for smoking, physical inactivity, and obesity, but men reported more often than women alcohol use (69.8% vs. 45.2%) and inadequate diet (77.9% vs. 66.7%). The highest prevalence of a single BRF among men was observed for alcohol use (81.1%) in the group with high level of education, for smoking (17.7%) and inadequate diet (83.0%) in the group with medium level of education. Among women, the most common BRFs were obesity (23.7%) among those with a low level of education, and physical inactivity (89.9%) among those aged 80 years and older.

Age and educational differences were seen for all BRFs, except for inadequate diet (Table 2). Younger participants reported alcohol use (60.6% vs. 47.0%), smoking (12.9% vs. 3.2%), and obesity (22.7% vs. 17.4%) more often than older participants, while older participants showed more often physical inactivity than the younger participants (88.5% vs. 73.8%). In sex-specific analyses, these age group differences were seen for smoking and physical inactivity in both sexes, for alcohol use in women only, and for obesity in men only. Compared with the highest level of education, those with a medium level of education reported smoking more often, and those with a low level of education reported physical inactivity and obesity more often. For alcohol use those with higher level of education reported alcohol use more often than both groups with lower education. In the sex-specific analyses, educational differences were seen for alcohol use in both sexes, for smoking in men only, and for physical inactivity in women only.

### Multiple behavioural risk factors

On average, participants reported 2.4 BRFs (95% CI: 2.3–2.4). Only 1.9% reported none; 15.0% one; and 83.1% multiple BRFs (Table 3).

**Table 3:** Prevalence of the number of behavioural risk factors in the total sample and by sociodemographic characteristics (%, 95% confidence intervals, weighted analyses)

	0	1	2	3	4	5	≥ 2
Total	1.9 (1.3–2.8)	15.0 (13.3–16.9)	36.6 (34.5–38.9)	37.5 (35.1–40.0)	8.2 (7.0-9.6)	0.8 (0.4–1.5)	83.1 (81.0–85.0)
Sex							
Women	2.5 (1.5-4.0)	**18.0** **(15.6–20.5)**	**40.1** **(36.9–43.4)**	**33.6** **(30.6–36.7)**	**5.7** **(4.2–7.7)**	**0.1** **(0.02–0.7)**	**79.6** **(76.7–82.2)**
Men	1.2 (0.7–2.2)	**11.4** **(9.0-14.4)**	**32.4** **(29.3–35.7)**	**42.2** **(38.7–45.8)**	**11.2** **(9.1–13.7)**	**1.6** **(0.8-3.0)**	**87.4** **(84.3–89.9)**
Age groups in years							
65–79	1.9 (1.2-3.0)	14.5 (12.2–17.2)	**33.9** **(31.0-37.1)**	38.6 (35.4–41.9)	**9.9** **(8.3–11.8)**	**1.1** **(0.6–2.1)**	83.6 (80.6–86.1)
80+	1.9 (1.2-3.0)	16.0 (13.7–18.6)	**42.6** **(39.9–45.4)**	35.1 (32.2–38.1)	**4.3** **(3.4–5.5)**	**0.08** **(0.02–0.3)**	82.1 (79.4–84.6)
Education level							
Low	1.8 (1.0–3.0)	15.5 (12.9–18.4)	39.0 (36.1–42.0)	36.7 (33.4–40.1)	**6.0** **(4.5–8.1)**	**1.0** **(0.5–2.2)**	82.8 (79.6–85.5)
Medium	2.5 (1.4–4.2)	14.0 (10.9–17.8)	34.5 (30.2–39.1)	37.2 (32.6–42.0)	**11.0** **(8.5–14.2)**	**0.8** **(0.3–2.2)**	83.5 (79.7–86.7)
High	1.3 (0.6–2.8)	15.2 (12.4–18.6)	33.4 (28.8–38.3)	40.8 (35.6–46.2)	9.3 (6.7–12.7)	**0.03** **(0.01–0.2)**	83.5 (79.9–86.5)

bold type = indicates differences between the categories of the respective sociodemographic characteristic based on non-overlapping 95% confidence intervals

Group differences in the distribution of multiple BRF were primarily evident by sex. In general, men had a less favourable risk profile than women. Multiple BRFs occurred more often in men (87.4%) than in women (79.6%). Compared to women, men reported having three, four, or five BRFs more frequently and one or two BRFs less frequently.

### Combinations of behavioural risk factors

Of the 32 theoretically possible BRF combinations, 29 were observed in the sample (Table 4). The fewest number of combinations (*n* = 25) was found in the older age group. The most common combination in the total sample was alcohol use + inadequate diet + physical inactivity (21.8%), followed by inadequate diet + physical inactivity (16.0%), and physical inactivity alone (8.3%). The first two combinations ranked first and second across all sex and age groups; although not always in the same order. Among women and the older age group, physical inactivity alone was the third most common combination. Among men and the younger age group, however, the third most common combination was alcohol use + inadequate diet. The three most common BRF combinations were present in approximately half of the total sample (46.1%), i.e. in 47.1% of the women, 49.9% of the men, 42.6% of the younger age group, and in 60.5% of the older age group.

**Table 4:** Prevalence of behavioural risk factors (BRFs) combinations overall and by sex, age groups (weighted analyses)

BRF combinations	Total (*n* = 3,139)	Women (*n* = 1,484)	Men (*n* = 1,655)	65–79 y (*n* = 1,337)	80 + y (*n* = 1,802)
None	1.9	2.5	1.2	1.9	1.9
Alcohol use (A) only	3.1	3.0	3.2	3.5	2.1
Smoking (S) only	0.1	0.1	0.1	0.1	0.0
Inadequate diet (D) only	3.2	3.5	2.8	3.9	1.6
Physical inactivity (I) only	8.3	11.2	4.9	6.6	12.1
Obesity (O) only	0.3	0.3	0.4	0.4	0.1
AS	0.4	0.5	0.3	0.5	0.0
AD	7.9	6.4	9.7	9.6	4.0
AI	8.1	8.2	7.9	7.5	9.3
AO	0.6	0.7	0.5	0.6	0.6
SD	0.1	0.1	0.2	0.2	< 0.1
SI	0.5	0.8	0.1	0.6	0.3
SO	0.0	0.0	0.0	0.0	0.0
DI	16.0	19.3	12.2	11.8	25.4
DO	0.9	1.2	0.6	1.1	0.4
IO	2.1	3.0	1.0	1.9	2.5
ASD	1.2	0.9	1.5	1.7	< 0.1
ASI	0.6	0.8	0.5	0.7	0.5
ASO	0.0	0.0	0.0	0.0	0.0
ADI	21.8	16.6	28.0	21.2	23.0
ADO	1.8	0.2	3.6	2.3	0.6
AIO	2.1	2.2	2.0	2.0	2.3
SDI	2.9	3.0	2.7	3.7	0.9
SDO	0.1	0.0	0.2	0.1	0.0
SIO	0.1	0.1	0.0	0.1	0.0
DIO	7.0	9.7	3.7	6.6	7.7
ASDI	2.9	2.2	3.8	3.6	1.3
ASDO	0.1	< 0.1	0.2	0.1	0.0
ASIO	0.0	0.0	0.0	0.0	0.0
ADIO	5.0	3.3	7.1	5.9	3.0
SDIO	0.2	0.2	0.1	0.3	0.0
ASDIO	0.8	0.1	1.6	1.1	0.1
Number of combinations	29	28	28	29	25

y = years, 0.0 = no observation in the sample

### Sensitivity analyses

In the total study sample (*n* = 3,694), the unweighted proportion of missing data ranged between 1.4% for smoking and inadequate diet to 9.0% for physical inactivity. Overall, 85.0% of participants provided complete information on all BRFs, 11.1% had only one BRF missing, 3.1% two BRFs missing, and 0.9% three BRFs or more missing. To assess the potential impact of using a complete-case analysis on the prevalence of single BRFs, we compared the results with those obtained by allowing one missing BRF and by using available-case analyses based on all valid observations for each BRF. The resulting weighted prevalence estimates differed only marginally across approaches, with differences generally not exceeding 0.5% and a maximum difference of 1.1% (Supplementary material, Table 1).

Of the 3,139 participants included in the complete-case analysis, 261 participated by proxy. Compared with the main analysis and excluding proxy-reported data, prevalence estimates among self-reporting participants were slightly higher for alcohol use, smoking, and slightly lower for physical inactivity, as well as for some subgroups for inadequate diet and obesity (Supplementary material, Table 2). The 95% CIs overlapped for all prevalence estimates.

## Discussion

BRFs are highly prevalent among older adults in Germany. In Gesundheit 65+, about nine out of ten men and eight out of ten women show multiple BRFs. In addition, men exhibit more modifiable BRFs, such as alcohol use and inadequate diet, than women. Social inequalities and age-related effects are also evident among adults aged 65 years and older, predominantly to the disadvantage of those with lower levels of education (except for alcohol use) and of younger age.

In line with previous findings [11, 15, 17, 18, 36], the most frequent BRFs are physical inactivity (78.4%) and/or inadequate diet (71.8%). Obesity (21.0%) and smoking (9.9%) are observed less frequently. In contrast to other studies [11, 17, 18, 36], we found higher prevalence of the BRF alcohol use (56.3%) and lower prevalence of obesity, mainly due to different BRFs operationalisations. Using BMI of ≥ 25 as the cut-off would have yielded a prevalence of 63.2% for overweight and an altered ranking of BRFs. Our stricter definition of BRF alcohol use, based on new national guidelines [25] classified any alcohol use in the past week as BRF. A higher prevalence of 75.5% would even have been reported if any alcohol use in the past year had been regarded as BRF alcohol use.

Comparable to their younger counterparts [13, 20, 21], men aged 65 years and older show less favourable combinations of BRFs than women in our study, with higher prevalence of single BRFs such as alcohol use and inadequate diet, a higher average total number of BRFs, and more frequent occurrence of three or more risk factors. BRFs differ by age and education, with generally higher BRF prevalence in the younger age group and those with lower or medium levels of education; except for inadequate diet. Physical inactivity is more common among the older compared to the younger age group. Likewise, those with highest level of education report BRF alcohol use more often than those with lower or medium levels of education. Thus, associations of BRFs with age as well as socio-economic status known from younger cohorts also apply to older adults [11, 17, 18, 22, 23, 36].

Notably, not all possible combinations of the five single risk factors were observed within our sample. A decline in variability of combinations is particularly evident among the older age group. In addition, the three most prevalent combinations are reported by almost half of the sample. Not surprisingly, these three combinations include different combinations of the most frequent risk factors: physical inactivity, inadequate diet and alcohol use. This is consistent with findings from other for middle-aged adults [37].

### Strengths and limitations

The present study, Gesundheit 65+, a nationwide population-based sample of adults aged 65 years and older, offers several strengths. First, through disproportional random sampling we included a substantial number of participants from the oldest age group, especially women with higher levels of education and men aged 80 years and older. The effects of our sampling design as well as those of non-response were corrected by applying weights and using survey procedures in statistical analyses. Second, we assume our data are less selective than those of other studies (e.g. [11, 17–20, 36]) because we used a tested mixed-mode study design tailored to older adults [38], with only one study-specific exclusion criterion (i.e. insufficient knowledge of the German language) unrelated to age, institutionalisation or health status. Participation was possible despite cognitive or severe physical limitations by consent from a legal representative, support from others or proxy participation. Although the resulting overall response rate of 30.9% was modest, it reflects the broad eligibility criteria of the study and was calculated according to the ‘Standards of the American Association for Public Opinion Research’ [28], providing a conservative and transparent estimate of study participation. Third, we applied the most recent health behaviour recommendations, most notably for alcohol use. Defining any actual alcohol use as a BRF might seem strict, however, it is justified by the reduced tolerance, higher prevalence of (multiple) chronic conditions [1], and interactions with medication in older age [26].

On the other hand, our study also faces limitations. First, the cross-sectional design of our data set precludes the analysis of longitudinal changes in BRFs. Nonetheless, studies show that the total number of BRFs measured at some (arbitrary) point in midlife predicate premature mortality 20 years later (e.g. [39]). Second, the self- or proxy-reports may be biased by social desirability or a lack of knowledge (e.g. not knowing or underreporting body weight [40]). Nevertheless, the utilisation of self-report remains essential for large population-based and representative surveys with less selection bias. Third, BRFs may still be underestimated. The BRF physical inactivity was assessed without considering WHO recommendation of two muscle strengthening trainings per week [33]. Concerning the BRF alcohol use, any heavy episodic drinking (i.e. occasionally drinking high amounts of alcohol) outside the past week was not considered. The BRF inadequate diet was limited to the frequency of vegetable and fruit intake. Stricter cut-offs (≥ 5 servings per day) would have increased prevalence. In order to reduce participation burden, Gesundheit 65 + did not cover all dietary aspects relevant in old age, such as adequate fluid or protein intake, a topic that should be addressed in future research. Moreover, the analyses conducted did not consider the lack of personal influence on dietary and exercise habits in nursing homes or among people requiring assistance. Additionally, the influence of functional limitations such as sensory or mobility limitations on BRFs were not considered. Fourth, there may also have been some overestimation of BRF prevalence. For example, the data collection period partly overlapped with COVID-19 containment measures. This concurrence suggests a potential reduction in physical activity and fresh vegetables and fruit intake [41]. However, data were collected from June 2021 to April 2022, covering different periods of the pandemic and seasons, limiting systematic bias. Furthermore, the WHO recommendation on physical activity with 150 min could be too strict for our sample [33]. It is important to note that the distinction between moderate and vigorous physical activity was not made in the study. Consequently, participants who met the minimum requirement of 75 min of vigorous activity may have been misclassified as being inactive. Fifth, the main analyses were based on complete cases, which is a relatively conservative approach to handling missing data but provides a consistent basis for analysing the total number and combinations of BRFs. In addition, we used self- and proxy-reported data. Sensitivity analyses for the prevalence estimates of single BRFs using alternative approaches to handling missing data and excluding proxy-report yielded very similar results, suggesting that the influence of missing data and proxy-report on the main findings was limited. Finally, the unweighted total score used in this study assigns equal weight to all reported BRFs, regardless of their actual risk for specific diseases or premature death. Further research is needed to develop a total score that is weighted according to disease or mortality risk.

## Conclusion

There is a strong need for interventions to reduce health risk behaviour and obesity in older adults. Such interventions should consider living and care situations e.g. [42], sex and social inequalities [43], and follow a life-course approach. Population-based interventions, such as sugar taxes, reducing the influence of the food industry, and or banning alcohol advertising, still lacking in Germany, are crucial. The obstructive influences of the food and tobacco industries on effective public health interventions must be curtailed [44]. Public health interventions should be intersectoral and foster healthy living environments for all. In addition, health education and individual interventions should be targeted, low-threshold, proactive and multi-behavioural, i.e. designed to address multiple BRFs. For the majority of individuals aged 65 years and older, the primary focus of prevention strategies should be on the promotion of a balanced diet, age-appropriate physical activity, and the reduction of alcohol use.

## Supplementary Information

Supplementary material 1.

## Data Availability

The authors state that some access restrictions apply to the data on which the results are based. The data set cannot be made publicly available because the informed consent of the study participants does not cover the release of the data. The data set underlying the results is archived at the Research Data Centre of the Robert Koch Institute and can be accessed by researchers upon reasonable request. The data can be accessed on site in the Secure Data Centre of the Research Data Centre of the Robert Koch Institute. Requests can be sent by e-mail to fdz@rki.de.

## Abbreviations

BMI: Body mass index
BRFs: Behavioural risk factors
CASMIN: Comparative Analyses of Social Mobility in Industrial Nations classification
CI: Confidence intervals
Gesundheit 65+: Study on Health of Older People
WHO: World Health Organization
