# Supplementary material for: Prevalence of behavioural risk factors and their combinations among older adults: cross-sectional results of the nationwide study Gesundheit 65+
Source: BMC Geriatr. 2026 Aug 12;26:1047. doi: 10.1186/s12877-026-08007-3 (PMC13471662; doi:10.1186/s12877-026-08007-3)
Supplement: Supplementary file 1 [file 12877_2026_8007_MOESM1_ESM.docx]

Supplementary Table 1. Weighted prevalence of single behavioral risk factors using different missing-data approaches by sociodemographic characteristics (%, 95% confidence intervals).

|  | **Alcohol use** | **Smoking** | **Inadequate diet** | **Physical inactivity** | **Obesity** |
| --- | --- | --- | --- | --- | --- |
| Total |  |  |  |  |  |
| Number of missings in BRF | 180 | 51 | 51 | 331 | 131 |
| Complete cases analysis (n=3,139) | 56.3 (53.8-58.8) | 9.9 (8.4-11.6) | 71.8 (69.3-74.2) | 78.4 (75.7-80.9) | 21.0 (19.0-23.2) |
| One missing BRF allowed | 55.6 (53.2-58.0) | 10.1 (8.7-11.8) | 71.7 (69.5-73.9) | 78.7 (76.0-81.1) | 21.7 (19.7-23.9) |
| Available-case analysis | 55.8 (53.4-58.1) | 10.1 (8.6-11.7) | 71.8 (69.6-74.0) | 78.6 (76.0-81.1) | 21.8 (19.8-23.9) |
| Women |  |  |  |  |  |
| Number of missings in BRF | 96 | 20 | 21 | 173 | 70 |
| Complete cases analysis (n=3,139) | 45.2 (41.4-49.1) | 8.7 (6.9-11.0) | 66.7 (63.2-70.1) | 80.8 (77.3-83.8) | 21.2 (18.4-24.2) |
| One missing BRF allowed | 44.8 (41.1-48.5) | 9.1 (7.2-11.4) | 66.9 (63.7-69.9) | 81.2 (77.8-84.2) | 22.0 (19.3-24.9) |
| Available-case analysis | 45.2 (41.5-48.9) | 8.9 (7.1-11.2) | 67.2 (64.0-70.2) | 80.9 (77.6-83.9) | 21.9 (19.3-24.8) |
| Participants aged 80+ years |  |  |  |  |  |
| Number of missings in BRF | 123 | 35 | 33 | 215 | 92 |
| Complete cases analysis (n=3,139) | 47.0 (43.3-50.7) | 3.2 (2.5-4.1) | 68.1 (65.0-71.1) | 88.5 (86.5-90.3) | 17.4 (15.3-19.8) |
| One missing BRF allowed | 46.5 (43.1-49.9) | 3.3 (2.6-4.1) | 68.9 (66.0-71.7) | 88.8 (86.8-90.5) | 17.7 (15.5-20.1) |
| Available-case analysis | 46.6 (43.2-50.0) | 3.3 (2.7-4.1) | 69.2 (66.3-71.9) | 89.1 (87.2-90.8) | 17.9 (15.8-20.3) |
| Participants with a low education level^1^ |  |  |  |  |  |
| Number of missings in BRF | 108 | 28 | 25 | 193 | 71 |
| Complete cases analysis (n=3,107)^1^ | 48.2 (44.7-51.8) | 8.6 (6.9-10.6) | 72.1 (68.7-75.3) | 80.8 (76.6-84.3) | 23.1 (20.3-26.2) |
| One missing BRF allowed | 47.8 (44.5-51.1) | 9.0 (7.3-11.1) | 72.5 (69.4-75.4) | 81.1 (77.1-84.5) | 24.0 (21.0-21.2) |
| Available-case analysis | 47.9 (44.6-51.2) | 9.0 (7.3-11.0) | 72.5 (69.5-75.3) | 80.9 (77.0-84.3) | 23.9 (21.0-27.0) |

Note: BRF = behavioral risk factors, ^1^32 individuals did not provide information on their level of education and were additionally excluded.

Supplementary Table 2. Weighted prevalence of single behavioral risk factors based on self- and proxy-reports and self-reports only by sociodemographic characteristics (%, 95% confidence intervals, complete-case analysis)

|  | **Alcohol use** | **Smoking** | **Inadequate diet** | **Physical inactivity** | **Obesity** |
| --- | --- | --- | --- | --- | --- |
| Total |  |  |  |  |  |
| Self- and proxy-reports (n=3,139) | 56.3 (53.8-58.8) | 9.9 (8.4-11.6) | 71.8 (69.3-74.2) | 78.4 (75.7-80.9) | 21.0 (19.0-23.2) |
| Self-reports only (n=2,878) | 58.9 (56.4-61.3) | 10.3 (8.7-12.3) | 71.8 (69.3-74.3) | 77.2 (74.2-79.9) | 21.0 (19.0-23.3) |
| Women |  |  |  |  |  |
| Self- and proxy-reports (n=3,139) | 45.2 (41.4-49.1) | 8.7 (6.9-11.0) | 66.7 (63.2-70.1) | 80.8 (77.3-83.8) | 21.2 (18.4-24.2) |
| Self-reports only (n=2,878) | 48.7 (44.7-52.6) | 9.2 (7.2-11.7) | 65.8 (62.2-69.3) | 79.0 (75.4-82.3) | 21.1 (18.1-24.4) |
| Participants aged 80+ years |  |  |  |  |  |
| Self- and proxy-reports (n=3,139) | 47.0 (43.3-50.7) | 3.2 (2.5-4.1) | 68.1 (65.0-71.1) | 88.5 (86.5-90.3) | 17.4 (15.3-19.8) |
| Self-reports only (n=2,878) | 52.3 (48.5-56.0) | 3.5 (2.7-4.6) | 67.8 (64.3-71.2) | 86.7 (84.3-88.8) | 16.6 (14.3-19.2) |
| Participants with a low education level |  |  |  |  |  |
| Self- and proxy-reports (n=3,107)^1^ | 48.2 (44.7-51.8) | 8.6 (6.9-10.6) | 72.1 (68.7-75.3) | 80.8 (76.6-84.3) | 23.1 (20.3-26.2) |
| Self-reports only (n=2,850)^2^ | 51.4 (47.8-55.0) | 9.1 (7.3-11.3) | 72.2 (68.7-75.5) | 79.1 (74.7-83.0) | 23.1 (20.1-26.5) |

Note: ^1^32 individuals did not provide information on their level of education and were additionally excluded, ^2^28 individuals did not provide information on their level of education and were additionally excluded.
